# An In-Depth Investigation of Eye Movement Profile of Dyslexic Readers Using a Standardized Text-Reading Aloud Task in French

**DOI:** 10.3390/bs16010018

**Published:** 2025-12-21

**Authors:** Antonin Rossier-Bisaillon, Julie Robidoux, Brigitte Stanké, Boutheina Jemel

**Affiliations:** 1 École d’orthophonie et d’audiologie, Université de Montréal, 7077, Avenue du Parc, Local 3001-1, Montréal, QC H3N 1X7, Canada; julie.robidoux@umontreal.ca (J.R.); brigitte.stanke@umontreal.ca (B.S.); 2 Centre de Recherche Interdisciplinaire en Réadaptation du Montréal Métropolitain (CRIR), 6363, Chemin Hudson, Bureau 061, Montréal, QC H3S 1M9, Canada; 3 Institut Universitaire sur la Réadaptation en Déficience Physique de Montréal (IURDPM), Pavillon Lucie-Bruneau, CIUSSS du Centre-Sud-de-l’Île-de-Montréal, 2275, Avenue Laurier Est, 2e étage, Montréal, QC H2H 2N8, Canada; 4 Laboratoire de Recherche en Neurosciences et Électrophysiologie Cognitive, Hôpital en santé mentale Rivière-des-Prairies, CIUSSS du Nord-de-l’Île-de-Montréal, 7070, Boulevard Perras, Montréal, QC H1E 1A4, Canada

**Keywords:** reading aloud, dyslexia, eye movements, eye-voice span, word length and frequency, sublexical decoding

## Abstract

(1) Background: Most eye-movement studies in dyslexia focus on silent reading in controlled laboratory settings. Yet, oral reading of standardized texts remains central for identifying this disorder. By combining eye-tracking with oral reading, we captured both fixation dynamics and eye–voice span (EVS) measures, offering a richer view of the processes underlying dyslexia. (2) Methods: We tested 10 adults with dyslexia and 14 controls as they read aloud an unpredictable diagnostic text in French. Analyses examined psycholinguistic effects of word length and lexical frequency on fixation probabilities, counts, and durations, alongside EVS measures. (3) Results: Compared to controls, adults with dyslexia read more slowly, made more errors, and showed atypical fixation patterns: persistent word length effects, reduced frequency effects, and diminished, unstable EVS. (4) Conclusions: Together, eye-movement and EVS findings converge on a key mechanism: adults with dyslexia continue to rely heavily on sublexical decoding. This reliance creates a processing bottleneck in oral reading, where difficulties in rapid word identification cascade into sounding-out behavior and disrupted eye–voice coordination.

## 1. Introduction

While children formally begin learning to read aloud in the early school years, for many, the journey into print starts much earlier—nestled into bedtime routines, listening to parents bring picture books to life. It is in these moments that the magic unfolds: printed letters map onto sounds, and these sounds blend together to form words. Oral reading serves as a natural path to literacy, as it consolidates letter–sound correspondence (cf. Kuhn et al., 2006, for a review) and integrates both reading and listening comprehension to support understanding (Kim et al., 2011; Schimmel & Ness, 2017). While most children rapidly develop strong reading skills, 5–10% experience persistent difficulties despite normal intelligence and cognitive functioning (Wagner et al., 2020). These challenges often remain resistant to even intensive instruction, with many individuals continuing to struggle into adulthood. In this context, oral reading provides a uniquely sensitive window into reading ability, making it a powerful tool for detecting, evaluating, and understanding reading difficulties in clinical and educational settings (Bouma & de Voogd, 1974).

Among the most prevalent and well-studied of these reading difficulties is developmental dyslexia. Developmental dyslexia (from now on “dyslexia”) is a heritable neurodevelopmental condition with a genetic component (Gilger et al., 1992; Giraud & Ramus, 2013; Snowling & Melby-Lervag, 2016), characterized by persistent and specific difficulties in acquiring fluent and accurate word reading and spelling, despite adequate instruction and age-appropriate learning opportunities (American Psychiatric Association, 2013; Carroll et al., 2025). According to the recent international consensus by Carroll et al. (2025), dyslexia primarily reflects difficulties in learning and consolidating written-word forms (orthographic lexical learning), typically associated with phonological processing weaknesses, but not reducible to a single deficit. Although its precise etiology remains debated, several neurocognitive models have been proposed (Hancock et al., 2017; Ramus, 2004). Neurobiological accounts point to atypical functioning of the magnocellular visual pathway (Stein & Walsh, 1997), impaired neural entrainment to the temporal structure of speech (Goswami, 2011), whereas cognitive models traditionally emphasize a core phonological processing deficit (e.g., Goswami, 2002; Hulme & Snowling, 1994; Lyon et al., 2003; Stanovich, 1988; Vellutino et al., 2004). Other approaches highlight additional visuo-attentional difficulties that may contribute to the disorder (e.g., Bertoni et al., 2019; Bosse et al., 2007). In a multifactorial perspective, Carroll et al. (2025) emphasize that dyslexia results from the combined and interacting influences of genetic factors, atypical neurodevelopment, and multiple cognitive vulnerabilities. These vulnerabilities may involve phonological processing, memory, attention, and visuospatial or visuo-attentional skills, and their specific contribution may vary across individuals. This multifactorial account underscores that dyslexia emerges through diverse developmental pathways, explaining why profiles of strengths and weaknesses differ across children.

Regardless of the underlying mechanisms, dyslexia typically manifests in school-age children as unexpected difficulties in basic literacy skills such as word identification, decoding, and spelling. In reading more opaque orthographies such as English or French, these challenges manifest in both a slower and less accurate reading aloud (cf. Reis et al., 2020, for a review). Because these foundational skills are impaired, children with dyslexia often struggle to develop higher-level abilities such as reading comprehension, written expression, and vocabulary (American Psychiatric Association, 2013; Barrouillet et al., 2007; Carroll et al., 2025; Lefebvre & Stanké, 2016; Peterson & Pennington, 2015).

Dyslexia is a lifelong condition with long-term consequences: affected individuals are at greater risk of anxiety, depression, and low self-esteem (Daniel et al., 2006; Humphrey & Mullins, 2002), and may face challenges in academic, professional, and social domains (Maughan et al., 2020). Nonetheless, some adults with dyslexia develop compensatory strategies that allow them to overcome their reading difficulties, with many achieving education at the university level (Horn et al., 1999; Wagner et al., 2020). Previous data suggests that adults with dyslexia will often rely more strongly on their world knowledge and the sentential context (Bruck, 1990), a larger vocabulary (Cavalli et al., 2016) or strong morphological awareness skills (Law et al., 2015; Lefèvre et al., 2023) to identify words. As a result, their difficulties may sometimes go unnoticed in adulthood, complicating timely identification and appropriate support.

The identification of dyslexia relies on a comprehensive assessment of reading and writing skills at both the word and text levels, alongside evaluations of underlying cognitive factors such as phonological awareness, rapid naming, working memory, and attention (Collette & Schelstraete, 2016). In French-speaking populations, standardized batteries assess reading through single words (e.g., Phonolec; Plaza et al., 2008), meaningful texts (e.g., Evalad; Pech-Georgel & George, 2011), or meaningless texts that remove contextual support typically used as compensatory cues by readers with dyslexia (e.g., L’Alouette; Lefavrais, 2005). Despite their differences, these tests share a key feature: they all require reading aloud, which provides the clinician with measures of accuracy (number and type of errors), speed (words per minute), and efficiency (words correctly read per minute). Reading aloud is thus central to the diagnosis of dyslexia, as it probes multiple stages of word decoding, phonological access, and speech planning that converge in reading fluency. Yet, beyond these endpoint measures, reading aloud also engages complex psycholinguistic processes unfolding in real time—processes that remain hidden when only accuracy and speed are considered.

### 1.1. Eye Tracking and Dyslexia: Psycholinguistic Factors

Eye-tracking techniques provide a powerful window into the mechanisms underlying dyslexic reading and hold promises for improving its identification. Since the advent of modern eye-tracking systems in the 1970s, extensive research has documented the distinct eye-movement characteristics of individuals with dyslexia. Compared to their age-matched peers, both children and adults with dyslexia tend to fixate on more words while reading (De Luca et al., 1999; Hatzidaki et al., 2011; Hawelka et al., 2010) make more fixations on these words (De Luca et al., 1999, 2002; Hatzidaki et al., 2011; Hawelka et al., 2010; Hawelka & Wimmer, 2005; Hutzler & Wimmer, 2004) and sustain these fixations for longer durations (De Luca et al., 1999, 2002; Hatzidaki et al., 2011; Hawelka et al., 2010; Hutzler & Wimmer, 2004; Luke et al., 2024; Razuk et al., 2018). Their saccadic behavior is also atypical, with shorter forward saccades (De Luca et al., 2002; De Luca et al., 1999; Hawelka et al., 2010; Luke et al., 2024; Razuk et al., 2018) and more frequent backward saccades, termed “regressions” (De Luca et al., 2002; Hatzidaki et al., 2011; Hawelka et al., 2010; Hawelka & Wimmer, 2005; Hutzler & Wimmer, 2004; Razuk et al., 2018). Importantly, these differences are now understood as consequences of the reading disorder rather than evidence of a primary oculomotor deficit (Rayner, 1998). Supporting this view, such atypical patterns disappear in non-linguistic tasks (Zoccolotti et al., 1999) and are not observed when individuals with dyslexia are compared to younger, reading-age-matched peers (Hyönä & Olson, 1995; Olson et al., 1991; Zoccolotti et al., 2005, 2009). In sum, the hallmark difficulties of dyslexia are mirrored in distinctive eye-movement patterns that reveal the increased cognitive effort required for reading.

Beyond descriptive eye-fixation patterns, more fine-grained analyses of eye-tracking data can uncover the mechanisms that make word identification more effortful for children and adults with dyslexia. In a seminal study, Hawelka et al. (2010) demonstrated that German-speaking adolescents with dyslexia showed persistent word length effects on gaze durations, effects absent in their age-matched peers. This finding indicates that the number of graphemes directly impacts processing speed, reflecting a stronger reliance on decoding. Supporting this interpretation, the dyslexia group skipped fewer words and made more refixations, suggesting that words were often identified by moving the gaze sequentially from grapheme to grapheme. Thus, word-level eye-movement metrics provide crucial insights into the mechanisms that underpin reading difficulties. In their study, Hawelka et al. (2010) argued that these patterns align with the dual-route cascaded (DRC) model of reading (Coltheart et al., 2001). According to the DRC framework, word recognition can proceed via two parallel pathways: a lexical route, in which the orthographic representation of a word in long-term memory enables rapid identification, and a sublexical route, involving grapheme–phoneme conversion to sound out unfamiliar or pseudo-words (Share, 2008). Dyslexic readers’ stronger word length effects suggest greater reliance on this sublexical route, in contrast to typical readers who benefit more from the lexical route. Converging evidence supports this view, as studies consistently report disproportionate word length effects on multiple eye-movement measures in slow or dyslexic readers (De Luca et al., 2002; Gerth & Festman, 2021; Hutzler & Wimmer, 2004). Furthermore, it is important to note that these specific findings come from eye-tracking studies conducted in transparent orthographies such as German and Italian (Seymour et al., 2003). In these writing systems, the consistency of grapheme–phoneme correspondences encourages readers—including those with dyslexia—to rely on smaller grain-size units during decoding, as proposed by the psycholinguistic grain-size theory (Ziegler & Goswami, 2005). By contrast, readers of opaque or semi-opaque orthographies such as English and French tend to rely more on larger grain-size units and lexical processing, which can attenuate or mask word length effects. Converging cross-orthographic evidence indicates stronger word length effects in transparent than in opaque orthographies, not only in reading accuracy and latency (e.g., Egan et al., 2019), but also in eye-movement measures such as gaze duration (e.g., Rau et al., 2014). At the same time, lexical frequency effects—reflecting the efficiency of accessing stored word representations—remain robust across orthographies (Inhoff & Rayner, 1986; Yap et al., 2012). Taken together, these findings indicate that readers of less transparent orthographies depend less on sublexical processing, showing a different balance between sublexical and lexical mechanisms.

### 1.2. Oral Reading and the Eye-Voice Span

Eye-tracking research shows that oral reading represents a supplementary load when compared to silent reading. Reading aloud has been associated with longer fixations and saccades, an effect present in children (Kim et al., 2011; Vorstius et al., 2014), adolescents (Krieber et al., 2017) and adults (Inhoff et al., 2011; Laubrock & Kliegl, 2015). This pattern can be associated with the additional processes involved in reading aloud. A meta-analysis of PET and fMRI research shows that following word identification, a complex network is recruited to activate overt articulation (Jobard et al., 2003; Price, 2012). At the word level, this additional speech planning process is accounted for in cognitive dual-route (Coltheart et al., 2001) and connectionist (Zorzi, 2010) models of oral reading by the activation of a phoneme output system in reading aloud. In the case of sentence reading, this overt articulation also involves the production of an adequate reading prosody (Godde et al., 2020). Most importantly for the present study, another specific requirement of oral reading lies in the dynamic coordination of eye and voice.

A more extensive approach in reading research consists of the joint recording and analysis of eye and voice signals during oral reading. Classic work from early eye-tracking research showed that the eyes usually move ahead of the voice, maintaining a steady eye-voice span—the temporal or spatial gap between when a word is fixated and when it is spoken (Buswell, 1922; Fairbanks, 1937; Levin & Kaplan, 1970). This delay can be quantified temporally—as the temporal eye-voice span (the time between fixating a word and articulating it)—or spatially—as the spatial eye-voice span (the number of characters the eyes move ahead before the fixated word is spoken). Efficient oral reading requires regulating this gap: the eyes must stay far enough ahead to buffer upcoming material, yet not so far as to overload memory. Readers adjust the span dynamically by pausing fixations or by programming regressions when the voice lags behind, and by moving ahead more quickly when articulation permits. This principle has been demonstrated in reading aloud (Inhoff et al., 2011; Laubrock & Kliegl, 2015) and rapid automatized naming (Gordon & Hoedemaker, 2016; Ziaka & Protopapas, 2025). Because articulation is slower than visual decoding, the voice ultimately constrains oral reading speed, making it systematically slower than silent reading for longer passages. As of now, only one study conducted in Italian children has directly examined eye–voice coordination in dyslexia. De Luca et al. (2013) reported a reading aloud eye-tracking study in which dyslexic eye movements double in ratio when compared to controls. They also reported a markedly reduced spatial eye-voice span in children with dyslexia compared to their peers. This tighter coupling of gaze and speech may reflect difficulties accessing orthographic representations in long-term memory or a reliance on grapheme-by-grapheme decoding, echoing above-mentioned word length effects of sub-lexical processing in readers with dyslexia. In contrast, neurotypical readers typically adopt a more advanced reading style with their eyes moving further ahead in the text. However, the eye-voice span has yet to be directly investigated in French-speaking populations as well as in adults with developmental dyslexia. Given the frequent refixations and prolonged fixations in dyslexia, it is reasonable to expect that the size of the eye-voice span would be compromised during continuous text reading in this population.

### 1.3. Eye Tracking in Oral Reading Assessment Tasks

In recent years, several authors have underscored limitations of earlier eye-tracking studies on dyslexia, many of which relied on tightly controlled laboratory paradigms. For example, in their influential study linking word-level effects to phonological decoding, Hawelka et al. (2010) employed carefully selected target words to examine frequency and length effects. While such approaches have been instrumental for theory, calls have emerged for research using standardized reading tasks with greater ecological and clinical relevance (Franzen et al., 2021; Premeti et al., 2025). Responding to this need, Franzen et al. (2021) profiled eye movements of English-speaking adults with dyslexia using the International Reading Speed Test (IReST), a standardized text available in 19 languages designed to approximate everyday reading. Their results replicated classic dyslexic signatures—more numerous and prolonged fixations—and further revealed a novel cost of line switching; individuals with dyslexia required additional adjustments after return sweeps. However, their analyses did not pursue word frequency or length effects—key factors that differentiate readers with dyslexia from their peers—and their exclusive focus on silent reading diverges from the oral reading tasks central to reading assessment. More recently, Premeti et al. (2025) compared French-speaking university students’ eye movements while reading two widely used diagnostic texts: Le Pollueur (Gola-Asmussen et al., 2011), a meaningful newspaper-style passage, and L’Alouette-R (Lefavrais, 2005), a meaningless text composed of grammatically and syntactically correct sentences, with both frequent and rare words and spelling traps designed to block contextual compensation strategies. As expected, both texts revealed lower accuracy and slower reading in dyslexia. Eye-tracking showed that while fixations were not longer, they were more numerous, with word length predicting fixation counts in both groups but word frequency modulating fixation duration only in controls. Yet, these statistical analyses relied on Spearman regressions of mean values, limiting sensitivity compared to item-level modeling. Moreover, no attempt was made to investigate eye–voice coordination, a critical dimension for understanding oral reading and its clinical assessment in dyslexia.

In summary, recent research on eye movements in dyslexia has increasingly emphasized “real-world” texts that approximate clinically relevant reading conditions. This approach is promising for testing which predictions from theoretical models and laboratory paradigms generalize to more ecological reading-aloud settings. Findings indicate that eye movement differences in dyslexia remain robust even under these conditions. However, only one study has specifically examined word-level effects of lexical frequency and word length (Premeti et al., 2025), and existing studies either focus on silent reading (Franzen et al., 2021) or analyze oral reading without considering the dynamic relationship between eye and voice—i.e., spatial or temporal eye-voice span (Premeti et al., 2025). Although silent reading has dominated the literature (Rayner, 1998), it is inherently limited for dyslexia research, as clinical identification relies heavily on oral reading. Reading accuracy—a key diagnostic marker in more opaque writing systems such as English or French—cannot be directly measured in silent reading. Therefore, oral reading tasks offer both experimental control—i.e., placing all readers under the same conditions—and richer data linking eye movements directly to speech, making them particularly valuable for investigating dyslexia in adulthood.

### 1.4. The Present Study

The goal of the present study was to examine a combination of eye movement and spatial as well as temporal eye–voice span measures in dyslexic participants’ reading using a standardized, clinically relevant task in French. Despite decades of research on dyslexic eye movements in laboratory settings, there has been limited work applying these methods to materials that are meaningful in a clinical context. In the present study, adult participants with and without dyslexia read L’Alouette, a text commonly used to assess reading skills in French (Cavalli et al., 2017; Champagne et al., 2024; Lefavrais, 2005; Premeti et al., 2025). Our analyses focused on two complementary dimensions of reading. First, we examined word-level properties (word length and lexical frequency) to evaluate the respective contributions of phonological decoding and orthographic word recognition in the prediction of eye movement patterns. Second, we investigated spatial eye–voice span (spEVS) and temporal eye-voice span (tEVS) to characterize how eyes and voice are coordinated during reading aloud, with particular attention to both group-level differences and intra-text variability.

In short, our study builds directly on several critical gaps in the literature: while previous work has shown that children with dyslexia exhibit reduced spatial eye–voice spans (De Luca et al., 2013), no study has simultaneously examined the span of temporal and spatial eye–voice coordination in continuous text reading by adult readers with dyslexia.

In line with this two-part analytical approach, our hypotheses were as follows. First, given the predominance of lexical word recognition in adult skilled readers of a semi-opaque writing system such as French, we expected these readers to present strong frequency-related effects and less evident word length effects. Conversely, French-speaking adults with dyslexia were expected to exhibit more pronounced word length effects compared to their peers, alongside reduced lexical frequency effects, reflecting a persistent reliance on sublexical smaller gain-size unit decoding. Second, we predicted that adult readers with dyslexia would show a shorter eye–voice span, indicative of difficulties in maintaining an optimal interval between visual and articulatory processes during reading aloud. Such a pattern would be consistent with sound-by-sound decoding in dyslexia and would further support the conclusions that their reading remains strongly anchored in sublexical processing.

## 2. Materials and Methods

### 2.1. Participants

Twenty-four participated in this study (6 males and 18 females). All were native speakers of French and had normal or corrected-to-normal vision. They were aged between 18 and 56 years old (*M*_age_ = 30 years old and 1 month, *SD*_age_ = 11 years and 9 months). Although a majority of participants were college or university students at the time of the study (*n* = 17), some of them (*n* = 7) were also employed in a variety of sectors (e.g., cultural, government, marketing, university professor). All subjects in this study had at least a college or university-level education. All participants signed a consent form, and the protocol received ethical approval from the Research Ethics Committee in Rehabilitation and Physical Disability (CERRDP) at the Center for Interdisciplinary Research in Rehabilitation of the Greater Montreal (CRIR). Of these participants, 10 had received a diagnosis of dyslexia (DD group; *M*_age-DD_ = 31 years and 10 months; *SD*_age-DD_ = 13 years and 2 months). The 14 control participants reported no history of reading difficulties (NT group; *M*_age-NT_ = 28 years and 10 months; *SD*_age-NT_ = 11 years). The two groups did not differ in terms of age (Welch’s *t*(17.29) = −0.59, *p* > 0.5, Hedge’s *g* = −0.25). Male-to-female ratio was 2 to 8 in the dyslexia group and 4 to 10 in the control group. Exclusion criteria included presenting an autism spectrum disorder, hearing impairment, Tourette syndrome, non-corrected visual impairment, oculomotor disorder, neurological disorders or any other speech or language disorders that could influence reading.

**Figure 1:**
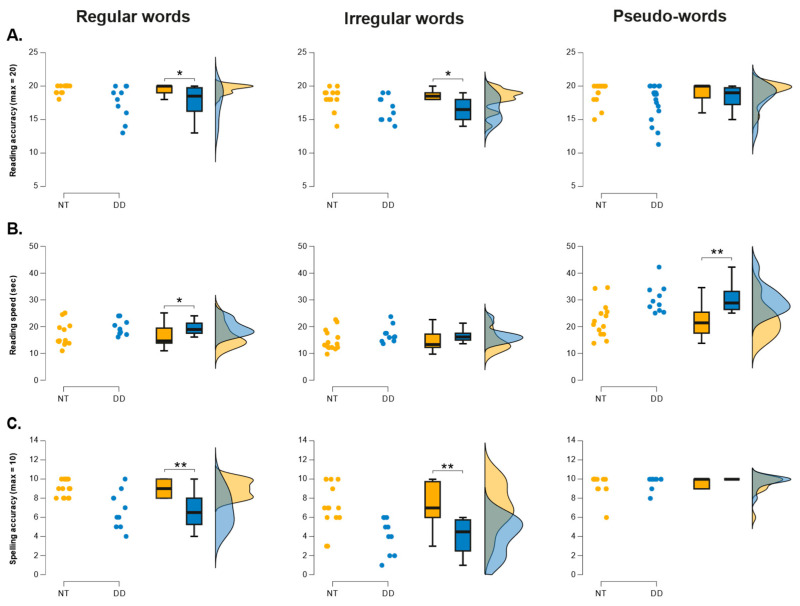
Raincloud plot of the performance of NT (in orange) and DD (in blue) participants in the ÉCLA-16+ (Gola-Asmussen et al., 2011)., a standardized test battery for French-speaking adults, assessing (**A**) reading accuracy, (**B**) reading speed, and (**C**) spelling accuracy for regular words, irregular words, and pseudo-words. Each dot represents an individual participant. Distributions are also represented to the right with boxplots and histograms. Brackets above graphs display significant group differences (*) when *p* < 0.05 and (**) when *p* < 0.005.

The reading proficiency and cognitive skills of the participants were assessed using subsections of ÉCLA-16+, a standardized test battery for French-speaking adults (Gola-Asmussen et al., 2011). Given the well-established directional impairments in reading and spelling observed in dyslexia, between-group comparisons were conducted using one-tailed Welch’s *t*-tests, consistent with our a priori expectation that the DD group would perform below the NT group. Complete descriptive statistics of the participants are presented in Figure 1. As can be seen in this Figure 1A, the DD group showed significantly lower accuracy than the NT group for regular (Welch’s *t*(9.83) = −2.393, *p* = 0.019, Hedge’s *g* = −1.024) and irregular word reading (Welch’s *t*(18.45) = −2.001, *p* = 0.030, Hedge’s *g* = −0.81), with no reliable difference for pseudowords (Welch’s *t*(9.83) = −1.074, *p* = 0.15, Hedge’s *g* = −0.442). For reading speed (cf. Figure 1B), the DD group exhibited slower latencies for regular words (Welch’s *t*(21.87) = 2.059, *p* = 0.004, Hedge’s *g* = 0.795) and pseudo-words (Welch’s *t*(21.48) = 3.254, *p* = 0.004, Hedge’s *g* = 1.279). The group difference for irregular words did not reach significance, although the effect size was moderate (Welch’s *t*(21.603) = 1.555, *p* = 0.067, Hedge’s *g* = 0.610; Figure 1B). Spelling accuracy was also lower in the DD group for both regular (Welch’s *t*(11.67) = −3.362, *p* = 0.003, Hedge’s *g* = −1.416; Figure 1C) and irregular words (Welch’s *t*(21.796) = −3.592, *p* < 0.001, Hedge’s *g* = −1.405; Figure 1C) with no reliable difference for pseudowords (Welch’s *t*(21.682) = 0.752, *p* = 0.77, Hedge’s *g* = 0.289; Figure 1C).

### 2.2. Apparatus

Eye movements were recorded using an Eyelink 1000 Plus eye tracker (SR Research Ltd., Kanata, ON, Canada) in a tower setup, which stabilized participants’ heads with forehead and chin rests. Viewing was binocular, but only the dominant eye was tracked (Miles, 1930), at a sampling frequency of 1000 Hz. Participants sat approximately 96 cm from a 24-inch ASUS VG248 LCD monitor (1920 × 1080 resolution, 144 Hz refresh rate). Oral reading responses were captured with a RØDE NTG1 microphone connected to an ASIO sound card (SoundBlaster Audigy 2-ZS, Creative Technology, Singapore) and digitized at a sampling rate of 24 kHz. The audio recording start time of the eye tracker was used to temporally align fixations with the audio track.

### 2.3. Reading Material

The reading material consisted of the Alouette-R text (Lefavrais, 2005), transcribed for on-screen presentation. All words were displayed in black on a white background, written in 24-point single-space Courier New font, so that a 1° of visual angle subtended approximately 3-character spaces. The full text is 265 words long. Because the full text could not be displayed in a single view while preserving a presentation format comparable to previous eye-tracking studies, it was divided into three passages that actually corresponded to the original three paragraphs of the text. The first passage contained 70 words across nine lines, the second 89 words across nine lines, and the third 106 words across twelve lines. The number of words per line ranged from five to sixteen. Lexical frequency values in written form were retrieved from Lexique 3, an online psycholinguistic database for French (New et al., 2004). Lexical frequencies (occurrences per million) ranged from 0.14 (e.g., *cordeaux*) to 38,928.92 (e.g., *de*), with a mean of 5401.01 (*SD* = 6506.89). Word length varied between 1 and 10 characters (*M* = 4.25; *SD* = 1.69).

### 2.4. Procedure

Each participant was tested individually in a quiet room with one or two experimenters present. Participants were instructed to read aloud three short passages that would appear sequentially on the display screen. They were asked to read at a normal pace and to minimize reading errors. Participants sat on a height-adjustable chair facing the computer monitor. Before the experiment began, a nine-point calibration procedure was performed, during which the eye tracker computed fixation positions based on the calibration. Calibration was considered successful if the average error was less than 0.5°. Following successful calibration, the three passages were presented one after the other. After the participant finished reading the final sentence of each passage, the experimenter pressed a button to terminate the display and advance to the next passage.

### 2.5. Data Preprocessing and Coding

Participants’ reading performance was assessed through both error counts and reading speed. Two raters from our team re-listened to the recorded .wav files of participants’ oral readings. With the text in hand, they transcribed verbatim reading errors as well as hesitations and self-corrections. Errors were defined as omissions and substitutions, while false starts and self-corrections were considered timing-related disruptions but were not scored as errors. This procedure allowed us both to quantify the number of errors and to obtain an accurate transcription of participants’ speech, which was subsequently required to automatically extract the onset and offset of each spoken word.

Eye-tracking data were preprocessed using the SR Research DataViewer software (SR Research Ltd., Kanata, ON, Canada). Fixations shorter than 50 ms were either merged with an adjacent fixation if the distance was less than 1°, or removed. Fixations longer than 1200 ms were also excluded. The following measures were analyzed: (1) Fixation probability measures: (a) word fixation probability (binomial measure), defined as the likelihood that a word was fixated during first-pass reading (0 = skipped, 1 = fixated); (b) number of first-pass fixations, defined as the total number of fixations on each word during the initial pass; and (c) regressive fixation probability (binomial measure), defined as the likelihood that a word was fixated via a regressive saccade (0 = not fixated regressively, 1 = fixated regressively). (2) Fixation time measures: (a) first-fixation duration, defined as the duration of the first fixation on a word, and (b) gaze duration, defined as the sum of all first-pass fixations on a word.

Eye–voice coordination measures, namely the temporal eye–voice span (tEVS) and the spatial eye–voice span (spEVS), were also computed. To achieve these measurements, audio files and transcriptions were first aligned using an automatic forced-alignment procedure, the Montreal Forced Aligner (McAuliffe et al., 2017), with a French pronunciation dictionary. The resulting TextGrid output was then inspected item by item with a customized MATLAB (Version 9.10) script to correct potential errors in the automated estimates of word onsets and offsets. Verification involved both auditory checks of the recordings and visual inspection of several displays, including an oscillogram, a spectrogram, and low-pass and high-pass filtered amplitude envelopes of the audio signal. From the corrected output, we extracted word-level onsets and offsets from the Alouette text to compute articulation durations. The tEVS corresponds to the time interval (in ms) between the onset of the first fixation on a word and the onset of its articulation. Positive values indicate that articulation followed fixation, with larger values reflecting a greater temporal lag between eye and voice. The spEVS was computed by determining the eye-gaze position at the onset of articulation of the target word and calculating the distance (in characters) between that position and the fixation point of the articulated word. A spEVS of zero indicates that gaze and voice were aligned on the articulated word; negative values reflect gaze lagging behind the spoken word, whereas positive values indicate gaze leading ahead. For both measures, missing values were assigned when no fixation occurred within the interest area of the articulated word.

### 2.6. Analyses

All analyses were conducted in R (Version 4.3.1; R Core Team, 2024) using RStudio (Version 2024.4.2.764; Posit Team, 2024). We first compared average reading performance and eye movement measures using Welch two sample *t*-tests. One-tailed tests were applied to evaluate directional hypotheses, grounded in longstanding theoretical and empirical evidence demonstrating predictable patterns of group differences between NT and DD individuals. Due to the small size of our NT and DD groups, we report effect sizes using Hedges’ g and its 95% confidence interval following Hedges and Olkin (1985). The magnitude of Hedges’ g corresponds to Cohen’s recommendations for interpreting effect sizes as small (0.2), medium (0.5), and large (0.8). Reading performance and eye movement measures were further analyzed with (generalized) linear mixed-effects models implemented in the lme4 (Version 1.1-38; Bates et al., 2015) and glmmTMB (Version 1.1.13; Brooks et al., 2017) packages. The fixed effects included a categorical predictor Group (NT vs. DD, with NT being the reference level), and two continuous variables, word length and word frequency, were first log-transformed (natural log for word length and base10 for word frequency) then zero-centered prior to analysis (e.g., Bell et al., 2009; for discussion; Kliegl et al., 2006; Kuperman & Bresnan, 2012; Yan et al., 2014). The reference level of these latter fixed effects is medium word length and medium frequency level, respectively. The random intercepts for both participants and items, and the random slopes for all the fixed effects, were included as random effects.

Participants and Words were treated as random effects, and Post hoc comparisons and simple slope analyses were performed using emtrends functions from the emmeans package (Version 1.10.6; Lenth et al., 2021). Interactions that involved continuous variables (e.g., word length, word frequency) were plotted using either simple linear regressions or using the ggpredict function from the ggeffects package (Version 2.3.0; Lüdecke et al., 2018). Binary outcomes (e.g., fixation probability: fixated vs. skipped) were modeled with logistic GLMMs, while zero-inflated or count data (e.g., number of fixations, revisits) were analyzed using negative binomial or zero-inflated models as appropriate. Models were fitted using the bobyqa optimizer with convergence criteria tightened (boundary tolerance = 0.001).

Our full model was represented as follows:

lmer(DV ~ group × WL\_center × WFreq\_center + (1 + WL\_center + WFreq\_center |Subj) + (1 |Word))(1)

Because the model failed to converge, we utilized a backward-stepping procedure to simplify the random-effects structure. This simplification involved removing the random slope of the main effects from the random structure (Barr et al., 2013). Our final BaseModel model was expressed as follows in lme4 syntax:

lmer(DV ~ group × WL\_center × WFreq\_center + (1 + WL\_center |Subj) + (1 |Word))(2)

Summary tables of all models, including significance values, effect sizes, and Akaike information criterion (AIC) were obtained from the tab\_model function from sjPlot package (Version 2.8.17; Lüdecke, 2021), and are reported in the Appendix A. Following recommendations from Meteyard and Davies (2020), we report standardized β coefficients (with SEs and 95% CIs) for all LMMs, as well as odds ratios (ORs) and incidence-rate ratios (IRRs) for logistic and count models, respectively. These scale-free effect-size measures allow for clearer comparison of effect magnitudes both within and across models and offer interpretable metrics for assessing underlying changes in probability or event rate. Standardized β coefficients reflect the change (in SD units) in the dependent variable associated with a one-SD change in a predictor. Larger absolute values indicate stronger effects and facilitate comparison across predictors measured on different scales. For logistic models, we provide odds ratios (ORs) and incidence-rate ratios (IRRs), respectively, as effect-size estimates. An OR or IRR of 1 indicates no effect, whereas Ors/IRRs above or below 1 reflect increases or decreases, respectively, in the odds of the outcome. The farther an OR/IRR deviates from 1, the larger the effect.

Finally, we compared the size of the temporal lag (tEVS) and spatial lag (spEVS) between the two groups, as well as the variability of these measures across participants. Variability was quantified using the coefficient of variation (cv), a standardized index expressing the ratio of the standard deviation to the mean. cv values approaching 1 indicate high variability (greater dispersion), whereas values approaching 0 reflect greater stability of the measure.

## 3. Results

### 3.1. Reading Performance Measures

**Figure 2:**
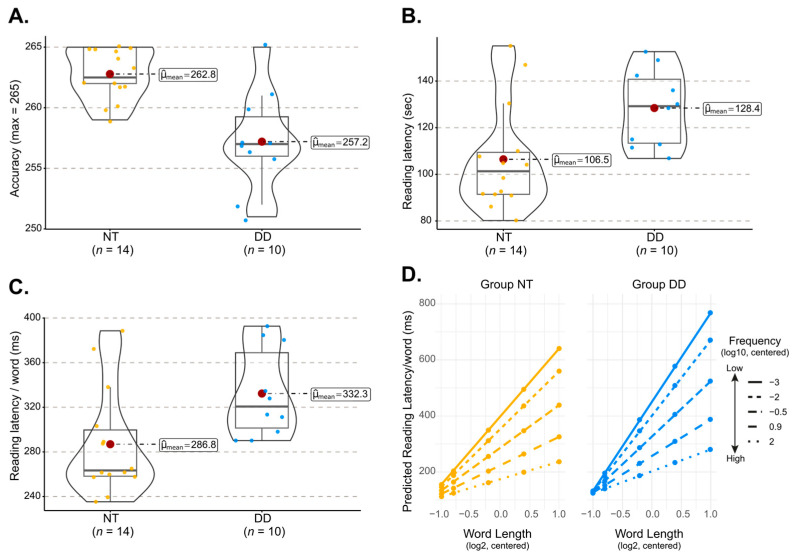
Violin plots showing reading performance results of NT (orange) and DD (blue) participants in terms of (**A**) reading accuracy, (**B**) reading speed, and (**C**) reading latency/correctly articulated word. The violin plot also shows means and standard deviations per group. Red dots represent means. Overlaid on each violin plot is a boxplot with a box ranging from the 25th to the 75th percentile, a line drawn at the median, and whiskers extending up to 1.5 times the interquartile range. (**D**) Results of LMMs for reading latency measure. Predicted reading latency per word of different levels of frequency (log10-transformed and centered) are plotted as a function of word length (log-transformed and centered), and separately for NT and DD participants.

As expected, participants in the DD group made significantly more errors (Welch’s *t*(12.44) = 3.95, *p* < 0.002; Hedge’s *g* = 1.75, 95% CI = [0.80, 2.67]; Figure 2A) and took significantly longer to read the Alouette-R text than their neurotypical (NT) peers (Welch’s *t*(21.97) = 2.756, *p* < 0.01, Hedge’s *g* = 1.04, 95% CI = [0.19, 1.88]; Figure 2B). In addition, reading times for individual words were prolonged in the DD group relative to the NT group (Welch’s *t*(21.38) = 2.54, *p* < 0.01; Hedge’s *g* = 0.98, 95% CI = [0.14, 1.81]; Figure 2C).

To further investigate group differences, we analyzed word-level reading times as a function of word length and frequency using a linear mixed-effects model (LMM; see Table A1). Reading times in NT participants increased with word length (*β* = 134.52, *SE* = 19.1, 95% CI = [97.09, 171.95]; *t* = 7.05, *p* < 0.001) and decreased with word frequency (*β* = −44.72, *SE* = 5.08, 95% CI = [−54.68, −34.78]; *t* = −8.8, *p* < 0.001), with the length effect particularly pronounced for low-frequency words (WL × WF: *β* =−36.13, *SE* = 7.1, 95% CI = [−49.98, −22.28]; *t* = −5.11, *p* < 0.001). A significant three-way interaction between group, word length, and frequency (*β* = −11.9, *SE* = 2.94, 95% CI = [−17.66, −6.15]; *t* = −4.05, *p* < 0.001) further revealed that the modulation of length effects by frequency differed across groups (see Figure 2D).

To unpack this interaction, we used the emtrends() function from the *emmeans* package (Version 1.10.6) to estimate word length slopes for each group across five levels of word frequency. A clear and systematic gradient emerged: group differences were largest for the lowest-frequency words and progressively diminished as word frequency increased. At very low frequency, DD participants exhibited substantially steeper word length slopes than NT participants *(Estimate_NT-DD_* = −75.2, *SE* = 19.7, 95% CI [−113.7, −36.65]; *z* ratio = −3.82, *p* < 0.0002). This large effect persisted at low frequency words (*Estimate_NT-DD_* = −63.28, *SE* = 18.39, 95% CI [−99.3, −27.23]; *z* ratio = −3.44, *p* < 0.001) and, although attenuated, remained reliable at medium frequency words (*Estimate_NT-DD_* = −45.43, *SE* = 17.27, 95% CI [−79.3, −11.55], *z* ratio = −2.63, *p* < 0.009). For higher-frequency words, group differences diminished and were no longer statistically reliable (|*Estimate_NT-DD_*| < 28.77; |*z* ratio| < 1.67, *p* > 0.095). These results indicate that the disproportionate length-related increase in reading latency among DD readers is concentrated on low-frequency lexical items, with group differences shrinking as word frequency increases.

We also examined how word frequency influences reading latency across five levels of word length by estimating word-frequency slopes for each group. A striking length-dependent pattern emerged. NT and DD groups showed comparable sensitivity to frequency for very short to medium length words (|*Estimate_NT-DD_*_|_ < 7.07; |*z* ratio| < 1.81, *p* > 0.07). However, the groups diverged sharply for long (*Estimate_NT-DD_* = 16.73, *SE* = 3.31, 95% CI [10.25, 23.21]; *z* ratio = 5.06, *p* < 0.0001) and very long words (*Estimate_NT-DD_* = 9.59, *SE* = 2.23, 95% CI [5.22, 13.96]; *z* ratio = 4.30, *p* < 0.0001): DD participants showed substantially steeper frequency slopes than NT participants. Together, these complementary trend analyses show that participants with DD experience the greatest difficulty reading long, low-frequency words, whereas group differences are reduced for short or high-frequency words.

### 3.2. Fixation Probability Measures

Here we examine group differences in three eye-tracking measures—word fixation probability, that is the probability of fixating a word among the 265 words in the text, during first-pass reading; number of first-pass fixations, and probability of regressive fixations—as a function of word length and frequency. Word fixation probability was coded binarily: words that were skipped during first-pass reading were assigned a score of 0, while words receiving one or more fixations were scored as 1. Regressive fixation probability, indicating whether a word was re-fixated via a backward saccade, was similarly coded as 0 (no regressive fixation) or 1 (at least one regressive fixation). First-pass fixation count reflected fixations on a word during its initial encounter.

**Figure 3:**
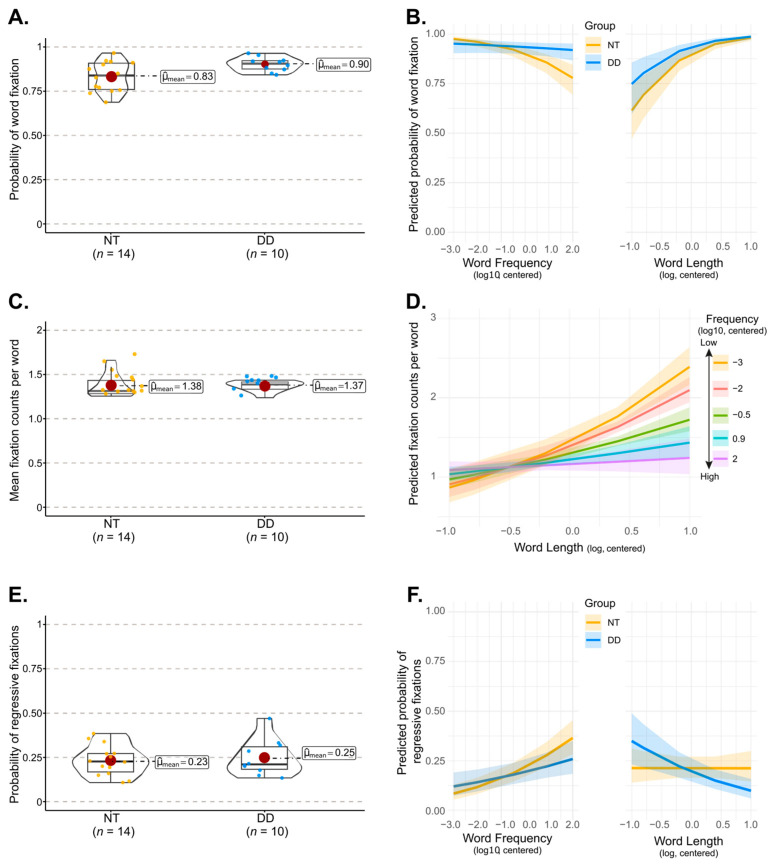
Violin plots showing (**A**) probability of word fixations, (**C**) fixation counts per word and (**E**) probability of regressive fixations for NT (orange) and DD (blue) participants. Red dots represent group means; violin plots display distributions with overlaid boxplots (25th–75th percentile, median line, whiskers at 1.5 × IQR). (**B**) Results of GLMMs for probability of word fixations showing a significant two-way interaction between group and word frequency (left panel) and non significant group × word length interaction (right panel). Predicted probability of word fixations for NT and DD participants are plotted by lexical frequency (log10-transformed and centered; left panel) and word length (log-transformed and centered; right panel). (**D**) Results of GLMMs for number of refixations on words showing a significant two-way word frequency × word length interaction. Predicted number of refixations, collapsed for NT and DD participants, are plotted by lexical frequency as a function of word length. (**F**) Results of GLMMs for probability of regressive fixations showing two-way group × word frequency (left panel) and group × word length interactions (right panel). Predicted regressive fixation probabilities for NT and DD participants are plotted by word frequency (**left**) and by word length (**right**).

As can be seen in Figure 3, overall DD participants fixated on significantly more words than NT participants (Welch’s *t*(19.72) = 2.667, *p* < 0.01; Hedge’s *g* = 0.96, 95% CI = [0.11, 1.78]; Figure 3A). However, they did not produce more fixations per word (Welch’s *t*(20.011) = −0.232, *p* = 0.598; Hedge’s *g* = −0.08, 95% CI = [−0.87, 0.70]; Figure 3C), nor did they make more regressive fixations (Welch’s *t*(17.32) = −0.395, *p* = 0.34; Hedge’s *g* = 0.16, 95% CI = [−0.62, 0.95]; Figure 3E) than NT participants. These global comparisons were further complemented by analyses examining how word length and word frequency jointly influenced these eye-tracking measures, and how these patterns differed between groups.

#### 3.2.1. Word Fixation Probability

These binary counts (0: skipped, 1: fixated) assessing word fixation probability were analyzed using Generalized Linear Mixed Models (GLMM) with a logistic link function, implemented via the lme4 package (Version 1.1-34.4; Bates et al., 2015) in R. We initially ran the analysis using the BaseModel syntax; however, the model failed to converge, requiring us to omit the random slope of participants by word length and the random intercept for item. The final model retained all fixed effects and interaction terms, with participant included as the sole random intercept (Table A2). Results showed that NT participants were more likely to fixate longer words (*β* = 1.8, *SE* = 0.24, 95% CI = [1.33, 2.26]; *z* = 7.63, *p* < 0.001), and less likely to fixate high frequency words (*β* = −0.49, *SE* = 0.07, 95% CI = [−0.64, −0.35]; *z* = −6.796, *p* < 0.001). These main effects were qualified by a significant word length × frequency interaction (*β* = −0.32, *SE* = 0.14, 95% CI = [−0.598, −0.05]; *z* = −2.32, *p* = 0.02). To probe this interaction, we conducted simple-slope analyses using the emtrends() function in R, testing whether each slope differed from zero. Word length robustly increased fixation probability at every frequency level (all *z* ratio > 5.10, *p* < 0.001). Frequency effects were also modulated by word length: for the shortest words, the frequency slope did not differ from zero (|*z* ratio| < 1.87, *p* > 0.062), whereas medium (OR = 0.764, 95% CI = [0.681, 0.857]) and long (OR = 0.695, 95% CI = [0.598, 0.808]) and the longest words (OR = 0.633, 95% CI = [0.493, 0.811]) showed significant negative frequency effects (all |*z* ratio| > 4.60, *p* < 0.001). Thus, frequency-related reductions in fixation probability were strongest for longer words.

Group differences emerged primarily in the effect of frequency (group × frequency: *β* = 0.385, *SE* = 0.12, 95% CI = [0.16, 0.61]; *z* = 3.32, *p* = 0.001; see Figure 3B). In contrast, the group × length interaction was not significant (*β =* 0.2, *SE* = 0.37, 95% CI = [−0.92, 0.822]; *z* = 0.53, *p* = 0.59; cf. Figure 3B). Slope of frequency effect compared to zero shows that in the NT group, higher frequency words were significantly less likely to be fixated than low frequency words (OR = 0.617, 95% CI = [0.536, 0.710], *z* ratio = −6.737, *p* < 0.0001; see Figure 3B), whereas in the DD group, the frequency effect was weaker (NT vs. DD: OR = 0.688, 95% CI = [0.549, 0.862], *z* ratio = 3.25, *p* = 0.0012) and not significant (OR = 0.617, 95% CI = [0.536, 0.710], *z* ratio = −6.737, *p* < 0.0001). The absence of a significant three-way interaction involving group, word length and frequency (*z* ratio = −1.6, *p* = 0.1) clearly indicates that this pattern did not vary with word length. Overall, NT participants were more likely to fixate longer and less frequent words than shorter and more frequent ones, whereas DD participants exhibited increased fixation probability only with word length only, and showed no reliable modulation by frequency.

#### 3.2.2. Fixation Counts per Word

To predict the number of first run fixations (discrete counts) as a function of group, word length and frequency, we ran generalized linear mixed-effects model using the glmmTMB package (Version 1.1.13; Brooks et al., 2017) for zero-inflated discrete data (Table A3). The effect of group was no significant (*β* = 0.001, *SE* = 0.03, 95% CI = [−0.074, 1.001]; *z* = 0.04, *p* = 0.97). However, both word length (*β* = 0.23, *SE* = 0.07, 95% CI = [0.099, 0.360]; *z* = 3.44, *p* = 0.001) and frequency were significant predictors (*β* = −0.05, *SE* = 0.02, 95% CI = [−0.09, −0.01]; *z* = −2.46, *p* < 0.015), and more importantly the interaction between word length and word frequency was significant (*β* = −0.08, *SE* = 0.02, 95% CI = [−0.13, −0.023]; *z* = −2.81, *p* < 0.005).

To unpack this interaction, we estimated the simple slopes of word length (tested against zero) at each frequency level, as well as the simple slopes of word frequency at each word length level (see Figure 3D). These analyses revealed that in NT participants, word length slope magnitude increased as frequency decreased: for the very high frequency words, the slope was essentially flat (OR = 1.066, 95% CI = [0.938, 1.211], *z* ratio = 0.977, *p* = 0.33) indicating that the number of eye-fixation did not increase with word length; however, the slope steepened progressively (OR > 1.177, *z* ratio > 3.035, *p* < 0.0025), reaching its maximum at the lowest frequency level (OR = 1.673, 95% CI = [1.428, 1.959]). Frequency slopes varied with word length as well: for short to medium-length words, fixation counts were largely unaffected by frequency (|*z* ratio| < 1.74, *p* > 0.08), with the corresponding slopes remaining essentially flat at a value consistent with one fixation on average (Figure 3D). In contrast, for longer words, the frequency slope became markedly steeper, indicating that lower-frequency words elicited reliably more fixations than high-frequency words (|*z* ratio| > 4.744, *p* < 0.001). The absence of any significant interaction involving group indicates that participants with DD displayed the same interaction pattern as NT participants (|*β*| < 0.029, *|z* ratio| < 0.65, *p* > 0.77).

#### 3.2.3. Probability of Regressive Fixations

All regressive fixations involved revisits to previously fixated words. To assess the probability of such word revisits, we submitted the binary outcome (0 = not revisited; 1 = revisited) to a GLMM with a logistic link function analysis. The initial BaseModel syntax specified the full-random effects structure; however, this model failed to converge. Following established recommendations for handling convergence issues in mixed-effects modeling (Barr et al., 2013), we simplified the random-effects structure in a stepwise manner. As for the fixation probability analyses, we first removed the random slope of word length for subjects, and subsequently removed the random intercept for items, resulting in a model with a random intercept for subjects only. This yielded a stable model whose results are reported in Table A4. Results show that, for the NT group, the likelihood of revisiting a word increased significantly with word frequency (*β* = 0.366, *SE* = 0.06, 95% CI = [0.25, 1.44]; *z* = 6, *p* < 0.001). In contrast, word length did not predict revisit probability (*β* = −0.004, *SE* = 0.195, 95% CI = [−0.39, 0.38]; *z* = −0.021, *p* = 0.98) and the word length × frequency interaction was not significant (*β* = −0.019, *SE* = 0.09, 95% CI = [−0.19, 0.98]; *z* = −0.22, *p* > 0.8).

Group effect emerged in the form of a significant word length by group interaction (*β* = −0.806, *SE* = 0.29, 95% CI = [−1.38, −0.23]; *z* = −2.74, *p* = 0.006), indicating that the impact of word length on word revisits differed between NT and DD participants. As can be seen in Figure 3F, the word length slope in the NT group was essentially flat, a pattern confirmed by simple-slope tests against zero (OR = 0.997, 95% CI = [0.678, 1.466], *z* ratio = −0.014, *p* = 0.99). In contrast, DD participants showed a reliably steeper negative slope, indicating a higher probability of revisiting shorter words than longer ones (OR = 0.451, 95% CI = [0.293, 0.697], *z* ratio = −3.595, *p* < 0.0001). Furthermore, the group × frequency interaction was marginal (*β* = −0.173, *SE* = 0.09, 95% CI = [0.006, 0.841]; *z* = −1.89, *p* = 0.059), suggesting a possible attenuation of the frequency effect in the DD group (see Figure 3F). Follow-up comparisons confirmed that although both groups showed a significant effect of frequency (*z* ratio > 2.76, *p* < 0.006), the slope was slightly but significantly reduced in DD (OR = 1.21, 95% CI = [1.06, 1.38]) relative to NT participants (OR = 1.44, 95% CI = [1.28, 1.62], *z* ratio > 1.96, *p* = 0.05). Finally, the three-way interaction between group, word length, and frequency was not significant (*β* = −0.204, *SE* = 0.13, 95% CI = [−0.46, 0.054]; *z* = −1.55, *p* > 0.12), indicating that the between-group differences in the pattern of word length and frequency effects were not modulated by a higher-order interaction.

### 3.3. Fixation Time Measures

**Figure 4:**
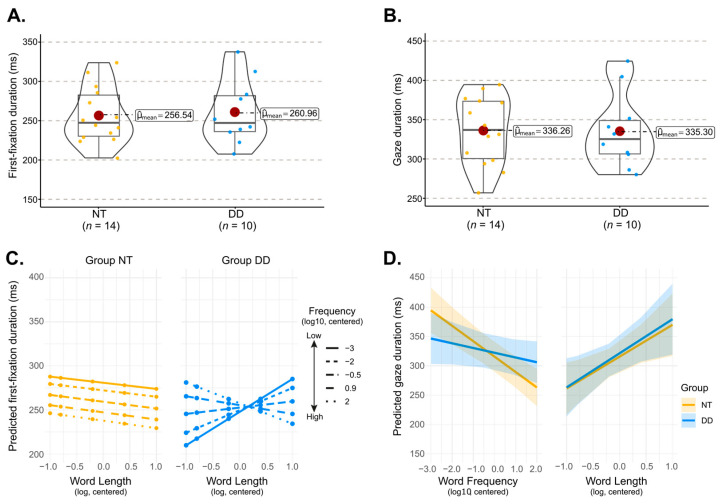
Violin plots showing (**A**) first-fixation durations (FFD) and (**B**) gaze durations (GD) for NT (orange) and DD (blue) participants. Red dots represent group means; violin plots display distributions with overlaid boxplots (25th–75th percentile, median line, whiskers at 1.5× IQR). (**C**) Results of LMMs for FFD showing a significant three-way interaction between group, word length, and word frequency. Predicted FFDs are plotted by word length (log-transformed and centered) and lexical frequency (log10-transformed and centered), separately for NT and DD participants. (**D**) Results of LMMs for GD showing a significant two-way interaction between group and word frequency (**left** panel) and no group × word length interaction (**right** panel).

In this section, we analyzed first-fixation duration (the duration of the initial fixation on the target word, regardless of subsequent fixations) and gaze duration (the sum of all first-pass fixations on the target word). As illustrated in Figure 4, DD and NT groups did not differ significantly in mean first-fixation duration (Welch’s *t*(17.75) = −0.273, *p* > 0.3, Hedge’s *g* = 0.11, 95% CI = [−0.67, 0.90]; Figure 4A) and gaze duration (Welch’s *t*(18.35) = 0.048, *p* > 0.9, Hedge’s *g* = −0.02, 95% CI = [−0.80, 0.76]; Figure 4B). To further examine the influence of word-level characteristics, we also ran linear mixed-effects models using the BaseModel syntax, testing the effects of word length and lexical frequency on fixation time measures in NT and DD participants.

#### 3.3.1. First Fixation Duration

Results of the linear mixed-effects analyses on first fixation durations are presented in Table A5. In NT participants, first fixation durations decreased as word frequency increased (*β* = −8.615, *SE* = 3.05, 95% CI = [−14.6, −2.63]; *t* = −2.821, *p* < 0.006), but were not affected by word length (*β* = −7.866, *SE* = 10.89, 95% CI = [−29.21, 13.48]; *t* = −0.722, *p* = 0.47), and the two factors did not interact significantly (*β* = −0.30, *SE* = 4.24, 95% CI = [−8.61, 8.00]; *t* = −0.071, *p* = 0.94). Although no overall group difference emerged (*β* = −1.38, *SE* = 15.221, 95% CI = [−31.22, 28.47]; *t* = −0.09, *p* = 0.93), a significant three-way interaction between group, word length, and word frequency was observed (*β* = −11.91, *SE* = 5.18, 95% CI = [−22.1, −1.76]; *t* = −2.30, *p* < 0.025), suggesting that the effect of word frequency and word length on first fixation durations in participants with DD differed from that in NT participants.

As illustrated in Figure 4C, the word length slope in the NT group was essentially flat across all frequency levels, a pattern confirmed by simple-slope tests against zero (|*Estimate*| < 8.47, *p* > 0.47). DD participants showed a similar pattern (|*Estimate*| < 23.43, *p* > 0.1) with one notable exception: for the lowest frequency words, the slope was reliably positive, indicating longer first fixation durations as word length increased for very low frequency words (*Estimate* = 37.64, *SE =* 19.1, 95% CI = [0.173, 75.1], *z* ratio = 1.969, *p* = 0.049). The effect of word frequency also varied as a function of word length and group. In the NT group, frequency slopes were negative for medium (*Estimate* = −8.55, *SE =* 3.25, 95% CI = [−14.92, −2.19]) and long words (*Estimate* = −8.74, *SE =* 3.35, 95% CI = [−15.3, −2.17]; both |*z* ratio| > 2.609, *p* < 0.01), and marginal for very long words (*Estimate* = −8.92, *SE* = 4.98, 95% CI = [−18.7, 0.84], *z* ratio = −1.791, *p* = 0.073). However, DD participants showed positive frequency slopes for very short and short words (*Estimate* > 11.82, *z* ratio > 2.231, *p* < 0.03), indicating longer fixations on higher frequency words at these lengths. The frequency slope then flattened at medium lengths (*Estimate* = 4.49, *SE =* 3.55, 95% CI = [−2.45, 11.5]; both *z* ratio = 1.3, *p* = 0.2) and gradually shifted toward a negative pattern as word length increased, converging with the NT pattern for very long words (*Estimate* = −10.17, *SE =* 5.43, 95% CI = [−20.8, 0.47]; both *z* ratio = −1.874, *p* = 0.061).

In the NT group, first fixation durations showed no reliable change with word length across frequency levels. In the DD group, first fixation durations were similarly stable except for low-frequency words, which showed a significant increase with word length. Word frequency reduced first fixation durations for NT participants at medium and long word lengths, whereas in the DD group, frequency increased first fixation durations for very short and short words before flattening and shifting toward negative values as word length increased, ultimately aligning with NT patterns for very long words.

#### 3.3.2. Gaze Duration

Results of the linear mixed-effects analyses on gaze durations are presented in Table A6. In NT participants, there were significant effects of word length (*β =* 53.316, *SE* = 21.596, 95% CI = [10.98, 95.66], *t* = 2.469, *p* < 0.016) and word frequency (*β* = −25.767, *SE* = 5.081, 95% CI = [−35.73, −15.81], *t* = −5.071, *p* < 0.0001), as well as a significant interaction between these two factors (*β* = −23.743, *SE* = 7.089, 95% CI = [−37.64, −9.84], *t* = −3.349, *p* < 0.001). Follow-up analyses of this interaction revealed that the negative frequency slope in NT participants was significant for medium to long words (*Estimate* > |11.60|, |*z* ratio| > 2.433, *p* < 0.015), with gaze durations reliably longer for low-frequency than high-frequency words at these word lengths; no frequency effect was observed for short words (*Estimate* < 8.59, *z* ratio < 1.061, *p* < 0.28). Word length effects were also modulated by frequency: increases in gaze duration with word length were present for words with medium and low frequency (*Estimate* > 67.79, *z* ratio > 3.672, *p* < 0.0002). A broadly similar pattern of effects was observed in participants with DD, consistent with the absence of a significant three-way interaction between Group, word length, and word frequency (*β* = −3.006, *SE* = 8.097, *t* = −0.371, *p* = 0.71). However, as illustrated in Figure 4D, while there was no significant interaction between group and word length (*β* = 3.09, *SE* = 28.31, *t* = 0.131, *p* = 0.89), the group factor interacted significantly with the word frequency factor only (*β* = 18.23, *SE* = 5.62, 95% CI = [7.21, 29.25] *t* = −3.24, *p* < 0.002). Analysis of estimated marginal trends revealed distinct slopes for the NT and DD groups for words with medium length: in NT participants, gaze durations decreased more steeply as word frequency increased (*Estimate* = −26.66, *SE* = 5.07, 95% CI = [−36.2, −16.32], *z* ratio = −5.18, *p* < 0.0001), whereas in DD participants, gaze durations showed virtually no frequency-related changes (*Estimate* = −8.09, *SE* = 5.44, 95% CI = [−18.8, 2.58], *z* ratio = −1.486, *p* = 0.13).

### 3.4. Measures of Eye and Voice Coordination

**Figure 5:**
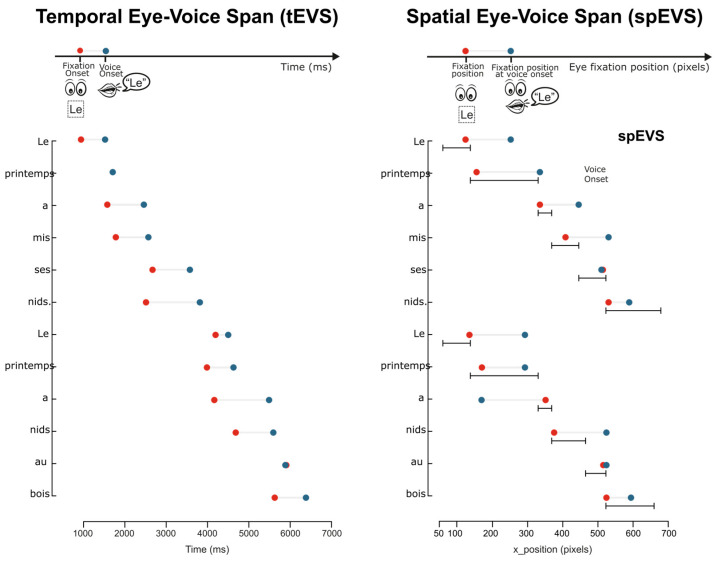
Illustration of the temporal (**left** panel) and spatial (**right** panel) eye-voice span (EVS) based on raw data from one participant (DD) reading two sentences form the Alouette text. In the left panel, the temporal EVS (tEVS) corresponds to the time lag between the onset of the eye fixation on a target word (red dot) and the onset of its articulation (blue dot). The gray line connecting the two events illustrates the magnitude of the temporal lag. In the right panel, the spatial EVS (spEVS) is shown as the distance between the eye position on the target word at fixation onset (red dot) and the eye position at the onset of articulation of that same word (blue dot). The gray line connecting the two points depicts the spEVS. Black horizontal bars indicate the start and end of each target word’s x-position on the screen.

Figure 5 presents sample data illustrating the temporal delay between the onset of fixation on a word and the onset of its articulation. This delay, measured in milliseconds, varies across the reading of sentences, as reflected by the differing lengths of the fixation–articulation intervals. As shown in the corresponding sample data in Figure 5 (right panel), the spatial EVS (spEVS) is computed by identifying the eye-gaze position at the onset of articulation and measuring the distance (in pixels, that is further transformed in number of characters) between that position and the fixation location on the articulated word.

**Figure 6:**
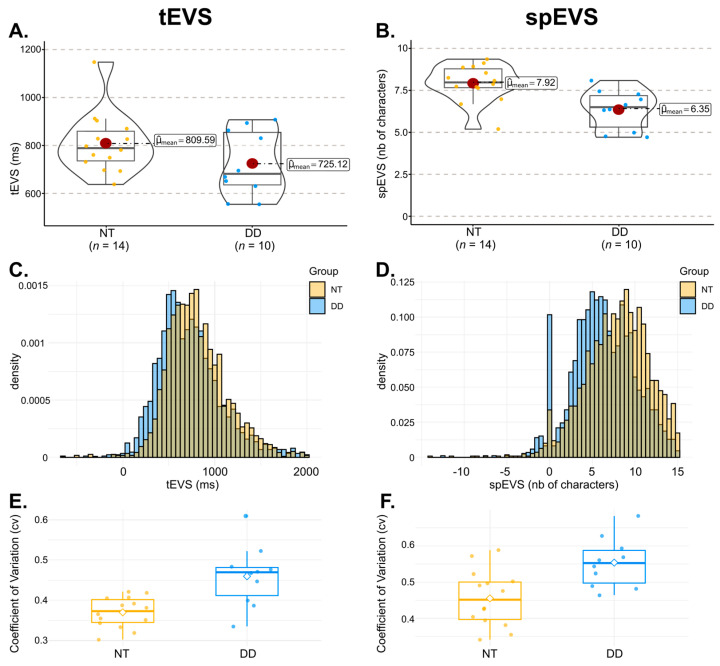
Violin plots showing (**A**) the temporal EVS (tEVS) and (**B**) the spatial EVS (spEVS) for NT (orange) and DD (blue) participants. Red dots represent group means; violin plots display distributions with overlaid boxplots (25th–75th percentile, median line, whiskers at 1.5× IQR). (**C**,**D**) respectively, display the probability density distributions (PDD) for tEVS and spEVS obtained from NT (orange) and DD (blue) participants. The bin width used for plotting tEVS was 50 ms, while for spEVS it was 1 character. Coefficients of variation for NT and DD groups are shown using boxplots for tEVS (**E**) and spEVS (**F**).

As illustrated in Figure 6A,C, the mean tEVS did not differ significantly between NT and DD participants (Welch’s *t*(18.56) = −1.542, *p* = 0.069, Hedge’s *g* = −0.63, 95% CI = [−1.42, 0.19]). However, group differences emerged for the spEVS measures (Welch’s *t*(18.71) = −3.289, *p* < 0.002, Hedge’s *g* = −1.33, 95% CI = [−2.19, 0.44]), indicating that the eyes had traveled a greater distance at the time of articulating the fixated word in the NT group compared to DD group (see Figure 6B). The density distribution plot in Figure 6D shows that, in a high proportion of instances, the gaze and voice were spatially aligned on the articulated word, as indicated by spEVS values equal to zero. To test whether this alignment occurred more frequently in DD participants than in NT participants, we calculated the proportion of words for which spEVS values fell within 0 ± 4 characters. Results of *t*-test confirmed that this alignment was indeed more frequent in DD participants than in NT participants (Welch’s *t*(14.13) = 3.32, *p* < 0.005, Hedge’s *g* = 1.43, 95% CI = [0.53, 2.31]). Moreover, when the eyes led ahead of the voice, the spEVS amplitude was significantly larger in NT than in DD participants (Welch’s *t*(18.24) = 2.85, *p* < 0.011, Hedge’s *g* = 1.16, 95% CI = [0.29, 2.00]). We further quantified the level of instability of the tEVS and spEVS using the coefficient of variation (cv). As can be seen in Figure 6E,F, participants with DD exhibited higher cv values than NT participants for both temporal and spatial EVS (Welch’s *t* > 3.28, *p* < 0.004, Hedge’s *g* > 1.28), indicating greater variability and less stable eye–voice coordination in the dyslexia group.

## 4. Discussion

The goal of the present study was to provide an in-depth examination of eye movements in French-speaking adults with dyslexia during oral reading of an ecologically relevant text frequently used in clinical evaluation (L’Alouette-R; Lefavrais, 2005). Unlike typical reading passages, this text is intentionally meaningless and unpredictable, thereby minimizing access to contextual or linguistic cues that might otherwise allow readers with dyslexia to compensate for their difficulties. Our analyses focused on two key dimensions: (i) the effects of word length and frequency on reading performance and eye-tracking measures, and (ii) both spatial and temporal metrics of the eye–voice span. This dual approach extends prior work on eye-movement profiles of dyslexia by applying it to a clinically grounded, naturalistic reading task conducted in French. In the following sections, we summarize and discuss our findings within the broader literature on eye-tracking and oral reading in dyslexia.

### 4.1. Reading Performance Measures

In terms of reading performance metrics, participants with dyslexia exhibited significantly slower reading speeds and reduced accuracy compared to controls. These findings align with Cavalli et al. (2018)’s, who demonstrated that the Alouette task is both highly sensitive and specific for distinguishing adults with dyslexia from typical readers. The combination of slowed processing and elevated error rates highlights the particular challenge of reading a meaningless text in which contextual or linguistic cues are unavailable to support word recognition. Frequent errors and spelling traps embedded in the Alouette-R (Champagne et al., 2024) likely contributed to increased hesitations and reliance on word-guessing strategies in the dyslexia group. This profile closely mirrors the behavioral outcomes reported by Premeti et al. (2025) in their eye-tracking study of French-speaking adults. Notably, in our study, the subtest where participants with dyslexia showed a particularly reduced reading speed relative to controls was the pseudoword reading task of the ÉCLA16+. Because many of the rare words in the Alouette-R resemble pseudowords, this parallel suggests that the two tasks impose comparable demands on sublexical decoding. Taken together, these results reinforce the validity of our sample and confirm that adults with dyslexia experience pronounced difficulties when confronted with rare or pseudo-words that cannot be recognized through orthographic memory or contextual support.

Since we identified the onset and offset of each spoken word to compute the eye–voice span, we were also able to take a step further in our analysis of reading speed by deriving a measure of word reading latency—the duration of individual word pronunciations, independent of inter-word pauses. LMMs revealed that participants with dyslexia produced significantly longer word reading latencies than their peers. This result indicates that their overall slower reading speed is not only due to hesitations or pauses between words, but also to the increased time spent articulating each word. Such a pattern is consistent with a greater reliance on sounding-out strategies, that is, assembling the word sound-by-sound or syllable-by-syllable before producing it as a whole, a well-documented hallmark of dyslexia (Hendriks & Kolk, 1997; Trenta et al., 2013). More fine-grained analyses clarified the source of this difficulty. Word reading latency was influenced by word length in both groups, as expected, but this effect was markedly stronger in dyslexia, particularly for low-frequency words. Thus, longer words disproportionately slowed reading in participants with dyslexia, especially when the words were rare or unfamiliar. This suggests that they relied heavily on grapheme-by-grapheme decoding rather than rapid lexical retrieval.

Taken together, these results confirm that adult readers with dyslexia were especially disadvantaged by the absence of contextual cues and word familiarity in the Alouette text. Their slower reading speed, reduced accuracy, and longer word reading latencies converge with the eye-movement and eye–voice span findings, pointing to the cascading impact of impaired word recognition on oral reading fluency in dyslexia.

### 4.2. Fixation Counts and Word Fixation Probabilities

The second set of results examined fixation probability, modeled as the likelihood of fixating a word versus skipping it. Both dyslexia and control readers showed the classic word length effect: longer words were more likely to be fixated (Rayner & McConkie, 1976; Rayner et al., 1996). In contrast, the two groups diverged in their sensitivity to word frequency. Controls were more likely to skip high-frequency words, whereas readers with dyslexia showed no such effect. This dissociation highlights a fundamental contrast between word length and frequency effects. Word skipping is known to rely heavily on parafoveal pre-analysis (Brysbaert et al., 2005; Drieghe et al., 2004, 2007; Rayner, 1998). In our data, word length effects indicate that both groups could extract low-level spacing information from the parafovea, enabling them to detect word boundaries and skip shorter words—a pattern consistent with prior research (Brysbaert et al., 2005). However, only controls were likely able to extract higher-level lexical information from upcoming words, allowing them to selectively skip frequent items, as typically observed in skilled adult readers (Rayner et al., 1996). In contrast, participants with dyslexia showed no sensitivity to frequency in parafoveal processing: regardless of how common a word was, they planned a fixation on it with equal probability. In short, while both groups accessed low-level spatial cues from parafoveal vision, only controls engaged in lexical pre-analysis, underscoring a key limitation in parafoveal processing in dyslexia.

Once a word was fixated, readers produced either a single or multiple fixations, captured by the measure of fixation count. Our analysis revealed that fixation count was jointly influenced by word length and word frequency: longer words were more likely to attract multiple fixations when they were also rare, whereas shorter words were unaffected by frequency. This interaction mirrors developmental patterns in which word length and frequency jointly shape eye-movement behavior during reading (Rau et al., 2014). More critically for our research question, there were no significant main effects or interactions involving group. This null result contrasts with Premeti et al. (2025), who, using the same Alouette task, reported a higher number of fixations per word in dyslexia—a finding interpreted as evidence of a strong sublexical component in dyslexic reading, with controls identifying words in a single fixation. Although we did not replicate this group difference, it is possible that evidence of sublexical processing in dyslexia manifested less in how often words were refixated and more in whether every word was fixated in the first place, regardless of its frequency or familiarity. We did not replicate previous findings showing group differences in global fixation counts across a wide range of word lengths and frequencies. This absence of a main effect may partly stem from our relatively small sample, which likely limited statistical power and prevented fixation count differences from reaching significance. Beyond sample-size constraints, the oral reading context may also have contributed: when reading aloud, eye movements must remain tightly synchronized with the voice, which can restrict natural variability in fixation patterns and reduce the sensitivity of fixation count as a discriminative measure (Inhoff et al., 2011; Kim et al., 2011; Krieber et al., 2017; Laubrock & Kliegl, 2015; Vorstius et al., 2014). Importantly, although we did not observe group differences in overall fixation counts, our results nonetheless revealed that evidence for sublexical processing in dyslexia emerged in a different form—namely, in the likelihood that each word was fixated at least once, regardless of its frequency or familiarity, rather than in how often words were refixated.

Measures of regressive fixation probability provide insight into both the unusual processing demands of the Alouette-R and the continued reliance on sublexical decoding in dyslexia. Regressive fixations, which index the need for verification or re-analysis, are typically more likely to target rare or unfamiliar words in natural reading, where lexical difficulty is a primary trigger. In the present study, however, even control readers showed a markedly different pattern: lexical frequency primarily determined whether a word was refixated, with frequent words more likely to be revisited. Participants with dyslexia exhibited a more complex pattern. In addition to showing a weaker lexical-frequency effect than typical readers, they were significantly influenced by word length, with shorter words more often revisited.

These findings are unexpected, given that highly familiar words rarely trigger regressions in conventional, meaningful texts. They also diverge from Premeti et al. (2025), who reported more regressions to longer words in dyslexia. A plausible explanation lies in the distinctive design of the Alouette-R. Although the text contains mostly high-frequency words, these words often appear in syntactically or semantically incoherent positions—such as *nid* (“nest”) used as a verb—or in contexts deliberately engineered to mislead readers toward close orthographic neighbors (e.g., *pompe* “pump” appearing where *pomme* “apple” would normally be expected). Thus, comprehension difficulty arises not from lexical rarity but from sentence-level incoherence, prompting readers to regress to familiar words in order to verify unexpected usages. However, this does not fully account for why regressions were more frequent for short words in the dyslexia group. This pattern becomes more interpretable when considering the fixation profile of the Alouette-R. As shown earlier, both short words and highly frequent words tended to receive only a single fixation—regardless of their lexical properties—making them relatively underprocessed during the initial pass. Consequently, these words disproportionately “benefited” from later regressions, particularly for dyslexic readers, who show reduced parafoveal extraction of lexical information (Luke et al., 2024; Rayner et al., 1989). If frequency cues are not efficiently processed in the parafovea, short words may not be sufficiently encoded before fixation, increasing the likelihood of subsequent regressions. This aligns with earlier findings on progressive fixation probability and with the broader pattern observed across analyses: dyslexic readers are consistently more influenced by word length, whereas NT readers rely primarily on lexical frequency.

Nonetheless, these interpretations should remain tentative. Regressive saccades are inherently variable and difficult to constrain experimentally (Rayner, 2009), emphasizing the need for carefully controlled follow-up studies to clarify the mechanisms driving these effects. Overall, while NT readers relied largely on lexical knowledge to guide regressions—even within this atypical text—readers with dyslexia were jointly influenced by word length and frequency, reflecting their continued dependence on small-grain, sublexical processing and a less efficient use of lexical cues.

### 4.3. First Fixation and Gaze Durations

Two measures of fixation duration were analyzed in the present study: first fixation duration (FFD), corresponding to the very first fixation on a word, independent of subsequent fixations, and gaze duration (GD), the sum of all first-pass fixations on a word. Consistent with Premeti et al. (2025) and Ward and Kapoula (2021), who also employed the Alouette-R text with adolescents with dyslexia, we observed no significant group differences in either measure. This finding is somewhat unexpected given prior literature documenting prolonged fixations in dyslexia (De Luca et al., 1999, 2002; Hatzidaki et al., 2011; Hawelka et al., 2010; Hutzler & Wimmer, 2004; Luke et al., 2024; Razuk et al., 2018). Several factors may explain this result. First, the Alouette-R text is highly unpredictable; even control participants encounter rare or novel words that can trigger extended fixations. Second, unlike laboratory paradigms using short words or sentences that directly probe immediate word recognition, a long and challenging text may attenuate differences between groups over the course of the task (see also, Premeti et al., 2025). Third, unlike many eye-tracking studies in children, our participants were adults with dyslexia, who may partially compensate in fine-grained eye movements for word recognition difficulties through greater reading experience, but still present greater delays in text reading taken as a whole.

Contrary to Premeti et al. (2025), our results of linear mixed-effects models incorporating text-level metrics with finer-grained psycholinguistic variables, such as word length and lexical frequency, alongside group factor on fixation durations, provided a more nuanced view of emerging group differences. Our analyses are better able to uncover subtle group processing differences that may remain hidden in coarse, aggregate measures, such as mean values for fixed levels of word frequency and word length (i.e., Premeti et al., 2025). We found that in control participants, word frequency only significantly predicted FFD: rare words elicited longer fixations, whereas word length had no effect. These results are consistent with previous eye-tracking literature, where frequency effects typically appear in FFD (Rayner, 1998, 2009), but word length effects are weak or absent (Hautala et al., 2011; Juhasz & Rayner, 2003; Vergilino-Perez et al., 2004). The absence of length effects in FFD is unsurprising, as this measure captures only the initial fixation; effects of word length usually emerge in gaze duration through subsequent refixations. In contrast, adults with dyslexia exhibited an atypical interaction between word frequency and word length. For high-frequency words, FFD decreased as word length increased, whereas for low-frequency words, longer words were fixated for more time than shorter ones. This pattern suggests two distinct processes: for high-frequency words, FFD decreased as word length increased, likely reflecting an immediate adjustment in which long words are rapidly re-fixated, as documented in earlier work (Kennedy, 2000). For low-frequency words, however, longer words elicited longer initial fixations likely indicating a prolonged initial attempt at lexical identification before a secondary fixation is executed at another location. Taken together, these results suggest that whereas controls’ first-fixation durations were driven purely by lexical frequency, dyslexic readers engaged in a more complex and less efficient process, possibly reflecting a heavier reliance on sublexical decoding strategies.

Subsequent analyses of GD reinforce the interpretation of FFD results by showing that readers with dyslexia rely more heavily on phonological decoding. GD, defined as the sum of all first-pass fixations on a word before a forward saccade, is particularly sensitive to word length because longer words typically attract more refixations (Vergilino-Perez et al., 2004; Vitu et al., 1990). Accordingly, GD is expected to increase with word length, especially for words that require effortful processing (Barton et al., 2014; Hyönä & Olson, 1995; Juhasz & Rayner, 2003; Kennedy, 2000; Rayner et al., 1996; Vitu et al., 1990). Control participants displayed the expected interaction between word length and frequency: high-frequency words showed no length effect, consistent with recognition through a single fixation, whereas low-frequency words elicited longer gaze durations when they were also long, reflecting sublexical decoding demands. By contrast, readers with dyslexia exhibited only a main effect of word length, with no modulation by frequency. This absence of frequency effects suggests a failure to activate stored lexical representations, leaving decoding primarily driven by grapheme–phoneme correspondences. Taken together, these findings indicate that while mean FFD and GD did not differ significantly between groups, lexical analyses reveal a fundamental divergence in processing strategies: controls flexibly recruit lexical access, whereas readers with dyslexia remain anchored to length-driven sublexical decoding.

### 4.4. Eye-Voice Span

A central feature of the present study was the use of a reading-aloud task to assess two metrics of eye–voice coordination in both groups. First, the temporal eye–voice span (tEVS) measures the interval in milliseconds between the onset of a word fixation and its articulation. While no significant group differences were observed in mean tEVS, readers with dyslexia exhibited markedly greater tEVS variability across the task, indicating less stable coordination between eye and voice. Second, the spatial eye–voice span (spEVS) reflects the number of characters between the gaze location at articulation onset, and the fixation point of the articulated word. Here, significant group differences emerged: adults with dyslexia showed reduced spEVS, as their eye-fixation location was often 0 to 4 characters away from the location of the spoken word. Their gaze tended to remain close to the word being articulated, whereas control readers’ gaze typically moved further ahead. As with tEVS, spEVS was more variable across the task in the dyslexia group, highlighting both a reduced and less consistent coordination between eye movements and speech.

In the present study, we investigated both tEVS and spEVS which uncovered distinct mechanisms of eye–voice coordination in the two groups. Notably, spEVS emerged as a more sensitive marker of dyslexic reading than tEVS, which showed largely comparable mean values across groups. This aligns with previous work in neurotypical adults demonstrating that eye–voice coordination is primarily regulated through spatial rather than temporal adjustments (Laubrock & Kliegl, 2015). In this context, for control readers, spEVS indicates that the eyes consistently move further ahead of the voice, reflecting their ability to pre-process multiple words via efficient lexical recognition, maintain these units in a phonological working memory buffer, and program the speech sequence. By contrast, previous studies conducted with children had shown that dyslexia was associated with a markedly reduced spEVS (in a half-size ratio), likely reflecting frequent sounding-out behavior that keeps the eyes closely coupled to the spoken word (De Luca et al., 2013). The present study is the first to examine this eye-voice span discrepancy in adults with dyslexia. Consistent with the developmental evidence, we found that adults with dyslexia also exhibited a reduced spEVS, with their eyes often anchored to the currently spoken word. These findings indicate that, even in adulthood, individuals with dyslexia continue to rely heavily on phonological decoding when reading unpredictable text. One interpretation is that enhanced demands on foveal processing in dyslexia may impede rapid lexical access and restrict parafoveal preview benefits, thereby constraining the extent to which eye movements can lead articulation (Meixner et al., 2022). Notably, however, the magnitude of the reduction we observed appears smaller than that documented in children. This attenuation suggests a degree of developmental compensation, with adults showing partial—but not complete—mitigation of the eye–voice coupling. Future research with larger and more age-diverse samples will be essential for tracing the trajectory of this compensatory development and for identifying the cognitive and linguistic factors that support improved eye–voice coordination across the lifespan.

One novel measure in the present study was the estimation of EVS variability in both modalities, measured via coefficient of variance. Interestingly, both tEVS and spEVS showed greater intra-text variability in the dyslexia group. In typical readers, variability in eye–voice span is dynamically managed via longer fixations or regressions (Gordon & Hoedemaker, 2016; Laubrock & Kliegl, 2015). In dyslexic readers, altered patterns of fixation probabilities, regressions, and fixation durations appear to undermine this online regulation, contributing to the observed instability. Reading aloud imposes strict temporal constraints that place continuous pressure on the multi-level reading pipeline (Jensen et al., 2021)—spanning parallel and serial processes from initial visual fixation, access to orthographic and lexical/semantic representations, phonological access, and articulation, while simultaneously previewing upcoming words. This implies that at any given moment, a disruption in one stage can derail the pipeline, forcing the reader to halt and re-engage, thereby creating a “processing bottleneck”. Taken together, these findings provide converging evidence that participants with dyslexia sample information sub-optimally and process it less efficiently, making their reading vulnerable to temporary failures. This cascade of inefficiencies can transform reading into a slow, effortful, and often frustrating experience for individuals with dyslexia.

Beyond their theoretical contribution, these findings open promising avenues for the development of more sensitive eye-tracking-based screening tools for dyslexia. Current machine-learning approaches often rely exclusively on raw or summary eye-movement metrics (e.g., Nerusil et al., 2021; Svaricek et al., 2025), yet rarely incorporate fine-grained lexical variables such as word length and word frequency—variables that proved essential here in distinguishing psycholinguistic processing profiles across groups. Moreover, current systems typically rely on eye-movement data alone. The present study demonstrates the added value of integrating synchronized eye and voice signals to compute eye–voice span measures, which capture essential aspects of visual–articulatory coordination and provide additional markers sensitive to dyslexia. Taken together, our findings—derived from a standardized reading task—underscore the importance of combining lexical-level information with multimodal eye and voice data. This integrated approach holds significant promise for the development of more accurate, efficient, and robust screening tools for dyslexia. From a clinical perspective, the present study highlights the value of integrating eye-tracking and synchronized voice recordings into the assessment of dyslexia in adults. Traditional clinical tools—such as timed oral reading tasks like L’Alouette-R (Lefavrais, 2005) or accuracy-based measures—capture important behavioral outcomes but provide limited insight into the moment-to-moment processes that give rise to reading difficulties. By contrast, eye-tracking offers an objective and fine-grained evaluation of visual and cognitive processing during reading, and the addition of eye–voice span measures further reveals how readers coordinate visual intake with articulation. For adults more specifically, this approach supports ongoing monitoring in professional or academic settings, helping to recommend accommodations like text-to-speech synthesis, or extended time—tailored to persistent deficits rather than assuming compensation with age. For instance, future clinical protocols could validate spatial eye-voice span as a biomarker for treatment efficacy.

### 4.5. Limits and Future Studies

This study is the first to examine in depth the effects of word length and word frequency on eye movement and eye-voice span measures in adults with dyslexia reading in French. A number of limitations nonetheless warrant consideration. First, the sample size was rather small compared to other studies in the field (e.g., *n* = 20 in each group in Premeti et al., 2025), which may have constrained statistical power for detecting between-group effects. To mitigate this, we reported effect sizes and confidence intervals for all key estimates, providing information beyond significance testing. Still, replication with larger samples is essential to confirm the robustness of the present findings.

Second, although our recruitment procedure matched groups on mean age and ensured proportional representation of participants in their 50s across both DD and NT groups, age remains a potential confound in reading research because eye-movement patterns can change across the adult life span (e.g., older adults often exhibit more frequent fixations and regressions; Kemper et al., 2004; Rayner et al., 2006). To minimize this risk we (i) matched groups on age during recruitment, (ii) tested age as a covariate, and (iii) verified that age did not interact with the key effects reported here. Nevertheless, future studies with more age-homogeneous samples may help isolate developmental or experiential contributions to reading behavior.

Finally, the use of the Alouette-R—a demanding, pseudo-content text—limits the generalizability of our results to more naturalistic reading situations. Nonsensical or highly difficult texts typically elicit a greater number of small progressive saccades (Premeti et al., 2025; Rayner et al., 2012), and thus may amplify certain eye-movement patterns. Although dyslexia-related differences have been shown to persist across texts of varying difficulty, including both the Alouette-R and meaningful passages (Premeti et al., 2025), the specific pattern of group differences reported here should not be directly generalized to other reading contexts. We observed an unexpected pattern of word revisits—NT readers revisited more frequent words, and DD readers revisited shorter words— both effects running counter to prior evidence linking revisits to long and/or low-frequency words. While this surprising result requires further investigation to clarify its underlying mechanisms, our findings nonetheless highlight fundamental distinctions in the predictors of eye-movement behavior across groups: word length emerged as the dominant driver in adults with dyslexia, whereas word frequency exerted a stronger influence in NT readers.

## 5. Conclusions

Taken together, our findings provide converging evidence for a persistent reliance on phonological decoding in adults with dyslexia—one that cascades into a constellation of consequences for eye-movement behavior and eye–voice coordination. This pattern is reflected in robust word length effects across fixation probabilities, regressions, and fixation durations, whereas skilled readers show weaker sensitivity to length and instead display strong lexical frequency effects on fixation behavior. The absence of such frequency effects in the dyslexia group may indicate difficulties accessing orthographic lexical representations, leading to a heavier dependence on sublexical, sound-based decoding. This interpretation is reinforced by their reduced spatial eye–voice span, which emerged as one of the most discriminative markers of dyslexia in our study: a constrained span suggests tightly coupled eye–voice coordination that limits the ability to look ahead in the text. Consistent with this profile, reading latencies were longer in the dyslexia group and were more strongly driven by word length, further pointing to sustained reliance on phonological assembly during reading aloud.

## Data Availability

The original contributions presented in this study are included in the article. Further inquiries can be directed to the corresponding author.

## Abbreviations

The following abbreviations are used in this manuscript:

GD	Gaze Duration
FFD	First Fixation Duration
tEVS	temporal Eye-Voice Span
spEVS	spatial Eye-Voice Span
LMM	Linear Mixed Model
GLMM	Generalized Linear Mixed Model
DD	Developmental Dyslexia
NT	Neurotypical

## Appendix A

**Table A1:** Results from the Linear Mixed Models with reading latency per word (ms) as dependent variable. Group (G), word length (WL) and word frequency (WF) as fixed effects. WL and WF were log-transformed and centered to obtain comparable estimates. Participants with WL and items were included as random slopes. The model was run on 6354 observations. Statistical significance of predictors was assessed using likelihood ratio tests (*p*).

Predictors	Std. *β* Coefficient	CI (95%)	*p*
(Intercept)	−0.23	−0.37–−0.09	**<0.001**
Group (DD)	0.21	0.02–0.40	**0.034**
Word Length (WL)	0.73	0.53–0.93	**<0.001**
Word Frequency (WF)	−0.24	−0.30–−0.19	**<0.001**
G × WL	0.21	0.03–0.40	**0.021**
G × WF	−0.03	−0.05–−0.00	**0.022**
WL × WF	0.20	−0.27–−0.12	**<0.001**
G × WL × WF	−0.06	−0.10–−0.03	**<0.001**
**Random Effects**			
σ^2^	5139.27		
τ_00_ (Item)	4757.02		
τ_00_ (Subj)	1820.42		
τ_11_ (WL|Subj)	1451.37		
ρ_01_ (Subj)	0.98		
ICC	0.57		
*N*_subj_	24		
*N*_Item_	265		
Observations Marginal R^2^/Conditional R^2^	6354 0.645/0.849		

AIC = 73,304.1.

**Table A2:** Results from the General Linear Mixed Models with word fixation probability as dependent variable. Group (G), word length (WL) and word frequency (WF) as fixed effects. WL and WF were log-transformed and centered to obtain comparable estimates. Solely participants were included as random intercept. The model was run on 4829 observations. Statistical significance of predictors was assessed using likelihood ratio tests (*p*).

Predictors	Odds Ratios	CI (95%)	*p*
(Intercept)	9.75	6.62–14.35	**<0.001**
Group (DD)	1.52	0.83–2.80	0.174
Word Length (WL)	6.02	3.80–9.55	**<0.001**
Word Frequency (WF)	0.61	0.53–0.70	**<0.001**
G × WL	0.82	0.40–−1.69	0.595
G × WF	1.47	1.17–1.84	**0.001**
WL × WF	0.72	0.55–0.95	**0.020**
G × WL × WF	1.40	0.93–2.10	0.110
**Random Effects**			
σ^2^	3.29		
τ_00_ (Subj)	0.11		
ICC	0.42		
*N*_subj_	24		
Observations Marginal R^2^/Conditional R^2^	4829 0.327/0.403		

AIC = 3161.3.

**Table A3:** Results from the General Linear Mixed Models (glmmTMB) with first-pass fixation counts per word as dependent variable. Group (G), word length (WL) and word frequency (WF) as fixed effects. WL and WF were log-transformed and centered to obtain comparable estimates. Participants with WL and items were included as random slopes. The model was run on 4829 observations. Statistical significance of predictors was assessed using likelihood ratio tests (*p*).

Predictors	Incidence Rate Ratios	CI	*p*
(Intercept)	1.27	1.21–1.33	**<0.001**
Group (DD)	1.00	0.93–1.08	0.971
Word Length (WL)	1.26	1.10–1.43	**0.001**
Word Frequency (WF)	0.95	0.91–0.99	**0.014**
G × WL	1.03	0.85–1.25	0.772
G × WF	1.02	0.96–1.08	0.582
WL × WF	0.93	0.88–0.98	**0.005**
G × WL × WF	0.97	0.90–1.06	0.515
**Random Effects**			
σ^2^	0.55		
τ_00_ (Subj)	0.00		
ICC	0.00		
*N*_subj_	24		
Observations Marginal R^2^/Conditional R^2^	4157 0.059/0.060		

AIC = 10,142.1.

**Table A4:** Results from the General Linear Mixed Models with regression probability as dependent variable. Group (G), word length (WL) and word frequency (WF) as fixed effects. WL and WF were log-transformed and centered to obtain comparable estimates. Participants by WL and items were included as random intercept. The model was run on 4159 observations. Statistical significance of predictors was assessed using likelihood ratio tests (*p*).

Predictors	Odds Ratios	CI	*p*
(Intercept)	0.28	0.21–0.37	**<0.001**
Group (DD)	0.89	0.56–1.41	0.619
Word Length (WL)	1.00	0.68–1.46	0.984
Word Frequency (WF)	1.44	1.28–1.63	**<0.001**
G × WL	0.45	0.25–0.80	**0.006**
G × WF	0.84	0.70–0.10	**0.059**
WL × WF	0.98	0.83–1.16	0.826
G × WL × WF	0.82	0.63–1.06	0.121
**Random Effects**			
σ^2^	3.29		
τ_00_ (Subj)	0.25		
ICC	0.07		
*N*_subj_	24		
*N*_Item_	205		
Observations Marginal R^2^/Conditional R^2^	4159 0.093/0.158		

AIC = 4238.

**Table A5:** Results from the Linear Mixed Models with first-fixation duration as dependent variable. Group (G), word length (WL) and word frequency (WF) as fixed effects. WL and WF were log-transformed and centered to obtain comparable estimates. Participants by WL and items were included as random intercept. The model was run on 4142 observations. Statistical significance of predictors was assessed using likelihood ratio tests (*p*).

Predictors	Std. *β* Coefficient	CI	*p*
(Intercept)	−0.02	−0.20–0.15	**<0.001**
Group (DD)	−0.02	−0.28–0.25	0.91
Word Length (WL)	−0.03	−0.12–0.06	0.47
Word Frequency (WF)	−0.2	−0.20–−0.04	**0.005**
G × WL	0.04	−0.07–0.15	0.48
G × WF	0.14	0.04–0.24	**0.005**
WL × WF	−0.002	−0.06–0.05	0.94
G × WL × WF	−0.08	−0.14–−0.01	**0.022**
**Random Effects**			
σ^2^	10,601.75		
τ_00_ (Item)	530.36		
τ_00_ (Subj)	1236.55		
τ_11_ (WL|Subj)	275.21		
ρ_01_ (Subj)	0.37		
ICC	0.15		
*N*_subj_	24		
*N*_Item_	205		
Observations Marginal R^2^/Conditional R^2^	4142 0.008/0.154		

AIC = 50,401.0.

**Table A6:** Results from the General Linear Mixed Models with gaze duration as dependent variable. Group (G), word length (WL) and word frequency (WF) as fixed effects. WL and WF were log-transformed and centered to obtain comparable estimates. Participants by WL and items were included as random intercept. The model was run on 4108 observations. Statistical significance of predictors was assessed using likelihood ratio tests (*p*).

Predictors	Std. *β* Coefficient	CI	*p*
(Intercept)	−0.10	−0.24–0.04	**0.16**
Group (DD)	0.03	−0.17–0.24	0.75
Word Length (WL)	0.14	0.03–0.25	**0.014**
Word Frequency (WF)	−0.23	−0.31–−0.14	**<0.001**
G × WL	0.01	−0.14–0.16	0.892
G × WF	0.16	0.06–0.25	**0.001**
WL × WF	−0.09	−0.15–−0.04	**<0.001**
G × WL × WF	−0.01	−0.08–0.051	0.71
**Random Effects**			
σ^2^	24,702.86		
τ_00_ (Item)	1861.63		
τ_00_ (Subj)	1665.64		
τ_11_ (WL|Subj)	2700.19		
ρ_01_ (Subj)	0.43		
ICC	0.14		
*N*_subj_	24		
*N*_Item_	205		
Observations Marginal R^2^/Conditional R^2^	4108 0.097/0.227		

AIC = 53,516.8.
